# Combined Effects of Elevated Temperature and Crude Oil Pollution on Oxidative Stress and Apoptosis in Sea Cucumber (*Apostichopus japonicus*, Selenka)

**DOI:** 10.3390/ijerph18020801

**Published:** 2021-01-19

**Authors:** Xishan Li, Chengyan Wang, Nan Li, Yali Gao, Zhonglei Ju, Guoxiang Liao, Deqi Xiong

**Affiliations:** 1 National Marine Environmental Monitoring Center, Dalian 116023, China; lxsdmu@outlook.com (X.L.); ln15542525989@163.com (N.L.); jzl621925@163.com (Z.J.); 2 College of Environmental Science and Engineering, Dalian Maritime University, Dalian 116026, China; monicachengyan@126.com (C.W.); xiongdq@dlmu.edu.cn (D.X.); 3 State Environmental Protection Key Laboratory of Coastal Ecosystem, Dalian 116023, China; 4 School of Marine Engineering, Jimei University, Xiamen 361021, China; yali_gao77@outlook.com

**Keywords:** crude oil, elevated temperature, sea cucumber, oxidative stress, apoptosis

## Abstract

Currently, global climate change and oil pollution are two main environmental concerns for sea cucumber (*Apostichopus japonicus*) aquaculture. However, no study has been conducted on the combined effects of elevated temperature and oil pollution on sea cucumber. Therefore, in the present study, we treated sea cucumber with elevated temperature (26 °C) alone, water-accommodated fractions (WAF) of Oman crude oil at an optimal temperature of 16 °C, and Oman crude oil WAF at an elevated temperature of 26 °C for 24 h. Results showed that reactive oxygen species (ROS) level and total antioxidant capacity in WAF at 26 °C treatment were higher than that in WAF at 16 °C treatment, as evidenced by 6.03- and 1.31-fold-higher values, respectively. Oxidative damage assessments manifested that WAF at 26 °C treatment caused much severer oxidative damage of the biomacromolecules (including DNA, proteins, and lipids) than 26 °C or WAF at 16 °C treatments did. Moreover, compared to 26 °C or WAF at 16 °C treatments, WAF at 26 °C treatment induced a significant increase in cellular apoptosis by detecting the caspase-3 activity. Our results revealed that co-exposure to elevated temperature and crude oil could simulate higher ROS levels and subsequently cause much severer oxidative damage and cellular apoptosis than crude oil alone on sea cucumber.

## 1. Introduction

Sea cucumber (*Apostichopus japonicus*, Selenka) is one of the typical marine benthic species, mainly inhabiting along the Northern Pacific coast (e.g., Russia, Japan, and northern China) [1,2]. For its high value in nutrition and pharmaceuticals, sea cucumber is considered as one of the most commercially valuable species among seafoods [3]. It has become one of the most numerous aquaculture species in China, with a total annual production of over 210,000 t in 2018 [4,5]. Generally, sea cucumber is mainly cultured by pond farming, pen culture, and sea ranching in the coastal zones of the Bohai Sea and the Yellow Sea of China [2,6]. However, these areas are often easily threatened by human activities (e.g., urban runoff, operational discharges, and offshore drilling) and seasonal environmental stresses (e.g., elevated temperature and low salinity) [2,6,7], leading to a huge economic loss in the aquaculture industry of sea cucumber. Moreover, sea cucumber is also an important marine benthic nutrient recycler and plays an essential role in marine ecosystem [8,9]. Once the growth and survival of sea cucumber or even their recruitment are grossly affected, it may directly impact the energy cycle of marine ecosystem and cause severe resource degradation in marine environment [10]. Furthermore, numerous studies have also suggested that sea cucumber, with high acute sensitivities to environmental changes, is a suitable candidate model organism for studying the effects of environmental stresses on marine benthos [5,11,12,13].

More recently, with the ever-increasing global production and consumption of crude oil, oil pollution derived from harbors, shipping, and offshore drilling could severely threaten marine ecological system and marine economy [14,15,16]. For instance, in July 2010, approximately 1500 t of crude oil was spilled from the Xingang Port of Dalian (China) into the Yellow Sea, leading to significant economic losses in the marine aquaculture industry in Liaoning Province, especially in sea cucumber culture [2]. From June to July 2011, a series of oil spill accidents (named the 2011 Bohai Bay Oil Spill) polluted over 840 km^2^ of sea area and caused direct economic losses up to CNY 12.56 billion in northern China [17]. Due to its limited or null motility, sea cucumber is generally under a greater threat from oil pollution than the migratory species with the ability to escape, such as fishes [2]. Besides, for recent several decades, global climate change has caused observable ocean warming due to carbon emissions from human activities [18]. With the high dependence of marine aquaculture on water temperature, ocean warming could severely threaten marine aquaculture ecosystem and production worldwide [19,20]. As the *Apostichopus japonicus* is a temperate species (optimal water temperature: 14–18 °C) and mainly cultured in shallow water, the influence of seasonal temperature fluctuations on sea cucumber could be much more obvious than other marine aquaculture species [21,22]. Hence, elevated water temperature (over 25 °C) in summer is one of the most major challenges for sea cucumber aquaculture. For instance, in July and August 2018, extremely high temperature (exceeding 32 °C) caused higher than 90% mortality of sea cucumber in many aquaculture ponds of northern China, especially for Shandong and Liaoning Peninsula, resulting in enormous economic losses [23,24]. Previous studies have reported that elevated temperature may alter the environmental distribution of oil and its biological effects on marine organisms, consequently intensifying the adverse impact on their survival or even population recruitment [25,26]. Taken together, elevated temperature and oil pollution have been considered as two main environmental concerns for sea cucumber culture. Much attention is necessary to explore the combined effects of elevated temperature and oil pollution on the physiological responses of sea cucumber.

Although crude oil comprises thousands of organic compounds, polycyclic aromatic hydrocarbons (PAHs) are generally known as the predominant toxic components of crude oil to marine organisms [27,28]. Several recent studies have reported that oil-derived PAHs could stimulate reactive oxygen species (ROS) production during their biotransformation, resulting in an elevation of ROS levels in marine organisms [2,29,30]. Overelevation of ROS levels could further induce oxidative damage of the biomacromolecules (including DNA, proteins, and lipids) [31,32], which is known as the primary mechanism involved in cell damage, apoptosis, and tissue injury in marine organisms exposed to oil-derived PAHs [29,33,34,35,36]. Additionally, numerous studies have documented that elevated temperature could cause physiological (e.g., increased oxygen consumption) and histological (e.g., tissue injury) changes in marine organisms, and it has been suggested that elevated temperature could also disturb the balance between endogenous and exogenous ROS levels and thereby cause an incapacity of the antioxidant defense system [23,37,38,39,40,41]. However, until now, only a limited number of novel studies have investigated the combined effects of elevated temperature and crude oil exposure on marine organisms, such as mahi-mahi (*Coryphaena hippurus*), polar cod (*Boreogadus saida*), and Atlantic cod (*Gadus morhua*) [26,42,43,44,45]. For example, mahi-mahi in early-life stages exposed to *Deepwater Horizon* (DWH) crude oil and elevated temperature (30 °C) showed significant differences in physiological responses, such as oxygen consumption, cardiac function, and overall survival, compared to exposure to DWH crude oil alone [26,42]. To the best of our knowledge, there is no available information on the combined effects of elevated temperature and crude oil pollution on sea cucumber to date. Therefore, in the present study, we exposed sea cucumber to elevated temperature (26 °C) alone, Oman crude oil WAF at an optimal temperature of 16 °C, and Oman crude oil WAF at an elevated temperature of 26 °C for 24 h. The ROS level, the capacity of antioxidant defense system, oxidative damage of the biomacromolecules, and cellular apoptosis were assessed to explore the effects of elevated temperature on the toxicity of crude oil to sea cucumber.

## 2. Materials and Methods

### 2.1. Experimental Design

Sea cucumbers *Apostichopus japonicus* (wet weight: 26.92 ± 13.95 g) were obtained from Dalian Pikou sea cucumber aquaculture zoning, Liaoning Province, China (39°27′ N, 122°25′ E), in June 2020. During an acclimatization period of 7 d, sea cucumbers were maintained in recirculating tanks containing around 60 L of pre-filtered natural seawater with a density of 15 per tank. The maintenance parameters were set as: Temperature 16.0 ± 0.5 °C, pH 7.9 ± 0.2, salinity 32.0 ± 1.0 psu, dissolved oxygen 7.1 ± 0.3 mg·L^−1^, and a photoperiod of 14 h light/10 h dark. Sea cucumbers were fed once with the formulated feeds each day during the acclimatization period.

**Figure 1:**
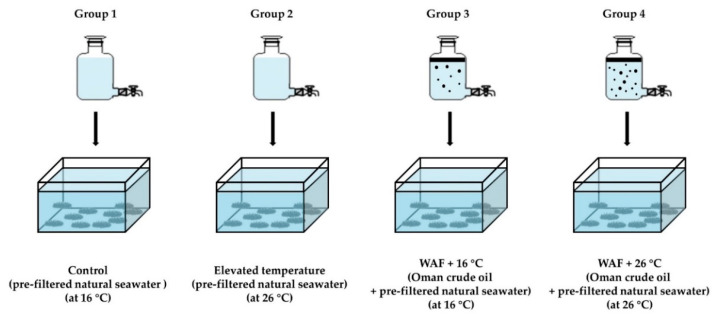
Experimental design of sea cucumber following exposure to elevated temperature (26 °C, Group 2), water-accommodated fractions (WAF) of Oman crude oil at an optimal temperature of 16 °C (WAF + 16 °C, Group 3), and Oman crude oil WAF at an elevated temperature of 26 °C (WAF + 26 °C, Group 4) for 24 h. The Control (Group 1) was the sea cucumber exposed to pre-filtered natural seawater only at an optimal temperature of 16 °C (*n* = 15).

After the acclimatization, sea cucumbers were randomly allocated to 4 treatment groups: 3 exposure groups (elevated temperature: 26 °C; Oman crude oil WAF at an optimal temperature of 16 °C: WAF + 16 °C; and Oman crude oil WAF at an elevated temperature of 26 °C: WAF + 26 °C) and 1 Control group (pre-filtered natural seawater only at an optimal temperature of 16 °C), as described in Figure 1 (15 sea cucumbers per group). Each group was replicated 3 times. WAF solutions of Oman crude oil (a light crude oil) were prepared on the basis of the methodology proposed by the Chemical Response to Oil Spills: Ecological Effects Research Forum (CROSERF) [46] with minor modifications [47]. Briefly, WAF solutions were prepared with Oman crude oil and pre-filtered natural seawater at an oil loading rate of 5 g·L^−1^ in 10 L glass aspirator bottles. The oil-seawater mixtures were mixed for 18 h and then settled for 6 h in the darkness. The WAF solutions were collected from the bottom of the aspirator bottles. The preparations of WAF solutions were performed in temperature-controlled rooms at 16.0 ± 0.5 °C and 26.0 ± 0.5 °C for WAF + 16 °C group and WAF + 26 °C group, respectively. All the solutions were freshly prepared prior to the exposure experiments. After an exposure period of 24 h, the body wall of each individual was dissected on an ice-cold Petri dish and washed with pre-cold 100 mM potassium phosphate (PBS) buffer (pH 7.4). Then, the tissue samples were frozen rapidly in liquid nitrogen and stored at −20 °C and then immediately used for the subsequent biochemical analysis. The animal experiments were reviewed by the Standardization Administration of China (SAC) GB/T 35823-2018 Laboratory animals-General requirements for animal experiment [48].

### 2.2. Analysis of Total Petroleum Hydrocarbons (TPH) and Polycyclic Aromatic Hydrocarbons (PAHs)

WAF solutions were stored at −20 °C in the darkness until chemical analysis. Total petroleum hydrocarbons (TPH) contents of WAF solutions were determined at 225 nm with the microplate ultraviolet-visible (UV-Vis) spectrophotometer according to the SAC GB 17378.4-2007 method [49] with minor modifications as described in our previous study [50]. In brief, 200 mL of WAF solution was extracted with 2 mL of H_2_SO_4_ solution (1:3, *v*/*v*) and 20 mL of *n*-hexane. The absorbance value of the extract solution at 225 nm was detected in a quartz cuvette by a microplate UV-Vis spectrophotometer (BioTek, Winooski, VT, USA). TPH concentrations of WAF solutions were calculated based on a standard curve of Oman crude oil (*r*^2^ = 0.991).

Additionally, the levels of United States Environmental Protection Agency (US EPA)’s 16 priority PAHs, including naphthalene (Nap), acenaphthylene (Acy), acenaphthene (Ace), fluorene (Fle), phenanthrene (Phe), anthracene (Ant), fluoranthene (Fla), pyrene (Pyr), benzo[*a*]anthracene (B[*a*]A), chrysene (Chr), benzo[*b*]fuoranthene (B[*b*]F), benzo[*k*]fuoranthene (B[*k*]F), benzo[*a*]pyrene (B[*a*]P), indeno [*1*,*2*,*3*-*cd*]pyrene (I[*123*-*cd*]P), dibenzo[*a*,*h*]anthracene (D[*ah*]A), and benzo[*g*,*h*,*i*]perylene (B[*ghi*]P) [51], were also analyzed according to the procedures as described in our previous works [47,52]. The pre-process of water sample (spiked with appropriate surrogate: 4-Terphenyl-d14) was performed based on the EPA 3510C liquid-liquid extraction method [53]. Then, the cleanup of the extract mixture was performed based on the EPA silica gel cleanup method [54]. Briefly, the extract mixture was dried with 4 g of granular anhydrous Na_2_SO_4_ and concentrated to 1 mL. After that, the concentrate was transferred into a silica gel column (containing 7 g of pre-activated silica gel and 1 g of Na_2_SO_4_). The collect from the silica gel column was concentrated, spiked with an internal standard mix (acenaphthene-d10, chrysene-d12, 1,4-dichlorobenzene-d4, naphthalene-d8, perylene-d12, phenanthrene-d10), and adjusted to 1 mL. Then, PAHs analysis was conducted by an Agilent 7890B gas chromatography (GC) coupled 5977A mass selective detector (MSD) (Agilent, Santa Clara, CA, USA) according to the ISO 28540:2011 method [55] with some modifications [47].

### 2.3. Reactive Oxygen Species (ROS) Level

ROS levels were detected according to the 2′,7′-dichlorodihydrofluorescein diacetate (DCFH-DA) method [56]. In brief, fresh body wall tissues were homogenized in pre-cold 100 mM PBS buffer at a ratio of 1:9 (*m*/*v*) using a D-130 handheld homogenizer (Wiggens, Straubenhardt, Germany). The tissue homogenates were centrifuged at 500× *g* for 20 min at 4 °C to collect the precipitate. Then, the precipitate was incubated for 60 min at 37 °C in 1.5 mL tubes, which contained 630 μL of PBS and 70 μL of DCFH-DA (1 mM). The fluorescence intensities of the samples were detected under 485 nm excitation and 525 nm emission by a SpectraMax M5 multimode microplate reader (Molecular Devices, San Jose, CA, USA). The ROS levels of each sample were normalized to the total protein (TP) content and expressed in arbitrary units per mg protein (AU·mgprot^−1^). The TP content of samples was determined at 562 nm via the microplate UV-Vis spectrophotometer based on the Bradford method [57].

### 2.4. Antioxidant Defense Capacity Assessment

To assess the capacity of antioxidant defense system, total antioxidant capacity (T-AOC) was measured in the present study based on the ferric reducing ability of plasma (FRAP) method [58]. Briefly, the body wall homogenates were centrifuged at 3000 rpm for 15 min at 4 °C to collect the supernate. Next, 4.1 mL of reaction volume (containing 200 μL of supernate) was mixed fully and then incubated for 10 min at 24 °C. The optical density (OD) of each sample was detected at 520 nm in a quartz cuvette with the microplate UV-Vis spectrophotometer. The T-AOC was normalized to the TP content of each sample and expressed in units per mg protein (U·mgprot^−1^).

### 2.5. Oxidative Damage Assessment

The level of oxidative DNA damage was determined using the 8-hydroxy-2′-deoxyguanosine (8-OHdG) enzyme-linked immunosorbent assay (ELISA) method [59]. In brief, 50 μL of the body wall body wall homogenate and 50 μL of biotinylated antibody working solution were added into a microtiter ELISA plate well (Nanjing Jiancheng Bioengineering Institute, Nanjing, China), which had been pre-coated with an antibody specific for 8-OHdG. The ELISA plate was incubated for 30 min at 37 °C. After the incubation, each well of the ELISA plate was washed 5 times with 300 μL PBS-Tween 20 (PBST) wash buffer. Then, 50 μL of avidin conjugated Horseradish Peroxidase (HRP) was added into each well of the ELISA plate. The ELISA plate was incubated again for 30 min at 37 °C and then washed 5 times with 300 μL PBST wash buffer. After washing, chromogenic solutions were added into each well and incubated for 10 min at 37 °C. Then, 50 μL of 2 M H_2_SO_4_ was added into each well to terminate the enzymatic color reaction. The OD value of each well was detected at 450 nm with the microplate UV-Vis spectrophotometer. The 8-OHdG level of each sample was calculated on the basis of a standard curve (*r*^2^ = 0.972), normalized to TP content, and expressed as ng per mg protein (ng·mgprot^−1^).

The level of protein oxidation was measured using the level of protein carbonyls (PCO), which was measured by the 2,4-dinitrophenyl hydrazine (DNPH) method [60]. Briefly, 100 μL of the body wall homogenate and 400 μL of 10 mM DNPH (in 2 N HCl) were mixed fully for 1 min and then incubated for 30 min at 37 °C in the dark. The reaction mixture was added with 500 μL of trichloroacetic acid (TCA), mixed fully for 1 min, and then centrifuged for 10 min at 12,000 rpm. The precipitate was washed 4 times with 1.0 mL of ethanol/ethyl acetate (1:1, *v*/*v*) mixture. The final precipitate was redissolved fully in 1.25 mL of 6 M guanidine hydrochloride and then incubated for 15 min at 37 °C. After incubation, the reaction mixture was centrifuged for 15 min at 12,000 rpm. The OD value of the final supernate was detected at 450 nm with the microplate UV-Vis spectrophotometer. The PCO level of each sample was normalized to TP content and expressed as nanomoles per mg protein (nmol·mgprot^−1^).

Moreover, the level of lipid peroxidation was also assessed in the present study based on the content of malondialdehyde (MDA) [61]. Briefly, 200 μL of the body wall homogenate, 200 μL of MDA Lysis buffer, 3 mL of phosphotungstic acid solution, and 200 μL of thiobarbituric acid (TBA) were added into a 5 mL tube and mixed fully. Then, the reaction mixture was incubated for 40 min at 95 °C, cooled in an ice bath, and centrifuged for 10 min at 4000 rpm to collect the supernate. The OD value of the supernate was detected measured at 532 nm with the microplate UV-Vis spectrophotometer. The MDA content of each sample was normalized to the TP content and expressed as nanomoles per mg protein (nmol·mgprot^−1^).

### 2.6. Apoptosis Assessment

The induction of cell apoptosis was evaluated according to the activity of caspase-3 [62]. In brief, 50 μL of the body wall homogenate, 50 μL of the Reaction Buffer (containing 10 mM DTT), and 5 μL of the 4 mM Ac-DEVD-*p*NA substrate were added into a 200 μL microcentrifuge tube and mixed well. Then, the mixture was incubated for 12 h at 37 °C. After incubation, the OD value of the mixture was detected at 400 nm with the microplate UV-Vis spectrophotometer. The relative change of caspase-3 activity of each treatment was expressed as the ratio of OD_(sample)_/OD_(negative control)_.

### 2.7. Integrated Biomarker Response (IBR) Index

To further compare the relative changes of biochemical markers in the different treatments (relative to the Control), the integrated biomarker response (IBR) indexes were calculated according to the methods as described by Beliaeff Benoit et al. [63] and modified by Sanchez Wilfried et al. [64] and Vieira et al. [65]. The deviations between different biochemical markers in different treatments (*X_i_*) were compared to those in the Control (*X*_0_). For each biochemical marker, the deviations (*X_i_*/*X*_0_) were log-transformed to *Y_i_* (Equation (1)). The general mean (*μ*) and standard deviation (*s*) of all *Y_i_* values were calculated. Then, *Y_i_* values were normalized (Equation (2)), and the difference between *Z_i_* and *Z*_0_ (the Control) was defined as the biomarker deviation index (*A*). The *A* value of each biochemical marker was calculated, and the absolute values of *A* were added up to obtain the IBR indexes for each treatment.

(1) $$Y_{i}=\log_{10}\frac{X_{i}}{X_{0}}$$

(2) $$Z_{i}=\frac{Y_{i}-\mu }{s}$$

### 2.8. Statistical Analysis

All the results were presented as the mean ± standard deviation (SD). Two-way analysis of variance (ANOVA) was performed to analyze the statistical difference among different treatments. Significant differences between different treatments were accepted when *p* < 0.05 and the asterisks *, **, or *** in the graphics denoted *p* < 0.05, 0.01, or 0.001, respectively. Data analysis and graph plotting were conducted using GraphPad Prism Ver 8.4 (GraphPad Software, San Diego, CA, USA).

## 3. Results

### 3.1. Analysis of Total Petroleum Hydrocarbons (TPH) and Polycyclic Aromatic Hydrocarbons (PAHs)

**Figure 2:**
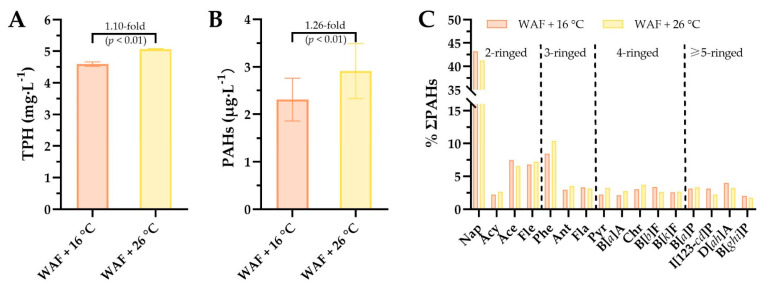
The concentrations of total petroleum hydrocarbons (TPH, (**A**)), the levels of US EPA’s 16 priority polycyclic aromatic hydrocarbons (ΣPAHs, (**B**)), and the proportion (%) of each PAH (**C**) in the water-accommodated fractions (WAF) of Oman crude oil at an optimal temperature of 16 °C (WAF + 16 °C, light-red filled) and Oman crude oil WAF at an elevated temperature of 26 °C (WAF + 26 °C, light-yellow filled) solutions. Nap: Naphthalene, Acy: Acenaphthylene, Ace: Acenaphthene, Fle: Fluorene, Phe: Phenanthrene, Ant: Anthracene, Fla: Fluoranthene, Pyr: Pyrene, B[*a*]A: Benzo[*a*]anthracene, Chr: Chrysene, B[*b*]F: Benzo[*b*]fuoranthene, B[*k*]F: Benzo[*k*]fuoranthene, B[*a*]P: Benzo[*a*]pyrene, I[123-*cd*]P: Indeno[1,2,3-*cd*]pyrene, D[*ah*]A: Dibenzo[*a*,*h*]anthracene, and B[*ghi*]P: Benzo[*g*,*h*,*i*]perylene.

Chemical analysis showed that TPH concentrations were 4.59 ± 0.07 mg·L^−1^ and 5.06 ± 0.02 mg·L^−1^ in the WAF solutions at 16 °C and 26 °C, respectively, manifesting that the TPH concentration in WAF solution at 26 °C was 1.10-fold higher than that in WAF solution at 16 °C (*p* < 0.01, Figure 2A). The contents of total 16 PAHs (ΣPAHs) were 2.31 ± 0.45 μg·L^−1^ and 2.91 ± 0.58 μg·L^−1^, respectively, indicating that the ΣPAHs content in WAF solution at 26 °C was 1.26-fold higher than that in WAF solution at 16 °C (*p* < 0.01, Figure 2B). Furthermore, we also analyzed the proportion (%) for each of the 16 PAHs as shown in Figure 2C. Results showed that the proportions (%) of 2-ringed, 3-ringed, 4-ringed, and ≥5-ringed PAHs in the WAF solution at 16 °C were 59.7%, 14.7%, 13.3%, and 12.3% of ΣPAHs, respectively. The proportions (%) of 2-ringed, 3-ringed, 4-ringed, and ≥5-ringed PAHs in the WAF solution at 26 °C were 57.6%, 17.0%, 14.9%, and 10.5% of ΣPAHs, respectively. Proportion analysis of these PAHs with different rings revealed that elevated temperature increased the proportion of higher molecular weight (HMW) (≥3-ringed), accompanied by a corresponding decrease in the proportion of lower molecular weight (LMW) PAHs (2-ringed).

### 3.2. ROS Level

**Figure 3:**
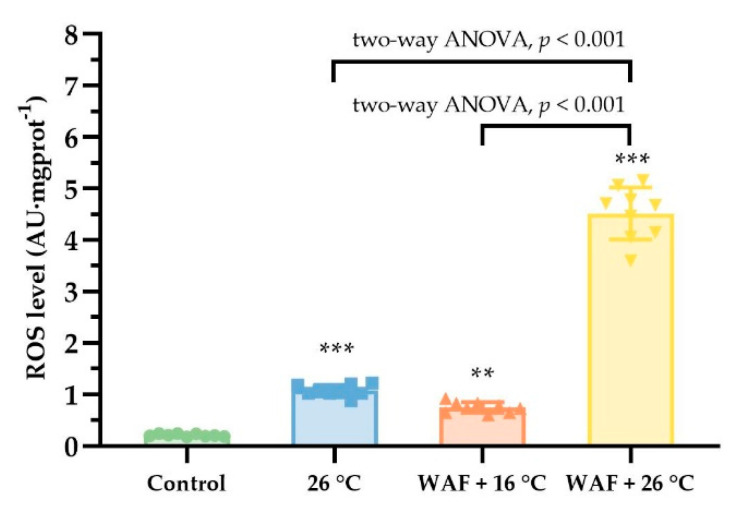
Reactive oxygen species (ROS) levels in the body wall of sea cucumber following exposure to elevated temperature (26 °C, light-blue filled square), water-accommodated fractions (WAF) of Oman crude oil at an optimal temperature of 16 °C (WAF + 16 °C, light-red filled up-triangle), and Oman crude oil WAF at an elevated temperature of 26 °C (WAF + 26 °C, light-yellow filled down-triangle). The Control was the sea cucumber exposed to pre-filtered natural seawater only at an optimal temperature of 16 °C (light-green filled circle). Error bars represent 95% confidence intervals. Asterisks (**, or ***) denote the significant differences between the treatments and the Control (*p* < 0.01, or 0.001, respectively). Dark traits denote the significant differences among the treatments (two-way analysis of variance (ANOVA)).

The ROS levels in the body wall of sea cucumber following different treatments are shown in Figure 3. The ROS levels in the 26 °C, WAF + 16 °C, WAF + 26 °C, and Control groups were 1.07 ± 0.11 AU·mgprot^−1^, 0.75 ± 0.10 AU·mgprot^−1^, 4.52 ± 0.48 AU·mgprot^−1^, and 0.21 ± 0.03 AU·mgprot^−1^, respectively, indicating that all treatments caused a significant rise in ROS levels compared to the Control (two-way ANOVA, *p* < 0.05). Moreover, the ROS level in WAF + 26 °C group was 4.21- and 6.03-fold higher than that in the 26 °C group and the WAF + 16 °C group, respectively, with significant differences (two-way ANOVA, *p* < 0.001).

### 3.3. Antioxidant Defense Capacity Assessment

**Figure 4:**
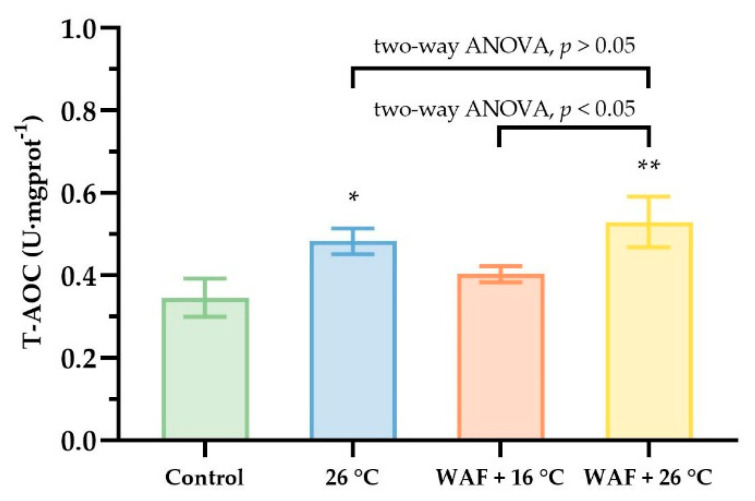
Total antioxidant capacity (T-AOC) in the body wall of sea cucumber following exposure to elevated temperature (26 °C, light-blue filled), water-accommodated fractions (WAF) of Oman crude oil at an optimal temperature of 16 °C (WAF + 16 °C, light-red filled), and Oman crude oil WAF at an elevated temperature of 26 °C (WAF + 26 °C, light-yellow filled). The Control was the sea cucumber exposed to pre-filtered natural seawater only at an optimal temperature of 16 °C (light-green filled). Asterisks (* or **) denote the significant differences between the treatments and the Control (*p* < 0.05 or 0.01, respectively). Dark traits denote the significant differences among the treatments (two-way analysis of variance (ANOVA)).

The T-AOC in the body wall of sea cucumber following different treatments are shown in Figure 4. The results showed that, compared to the Control (0.35 ± 0.04 U·mgprot^−1^), WAF + 16 °C treatment had no significant impact on the T-AOC (0.40 ± 0.02 U·mgprot^−1^; two-way, *p* > 0.05). Both 26 °C and WAF + 26 °C treatments caused a significant increase in the T-AOC (0.48 ± 0.03 and 0.53 ± 0.05 U·mgprot^−1^, respectively) relative to the Control (two-way, *p* < 0.05 and *p* < 0.01). Additionally, WAF + 26 °C treatment had a significant higher T-AOC than 26 °C treatment but without significant differences (two-way ANOVA, *p* > 0.05).

### 3.4. Oxidative Damage Assessment

**Figure 5:**
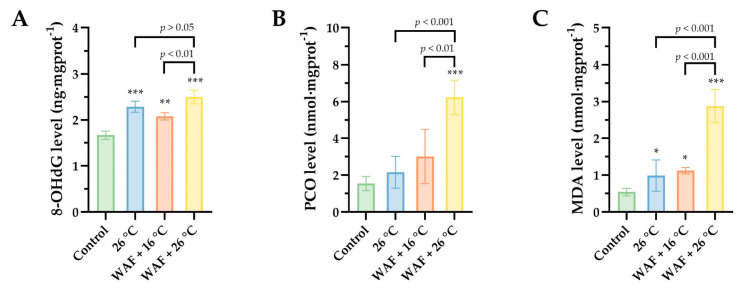
The levels of 8-hydroxy-2′-deoxyguanosine (8-OHdG) (**A**), protein carbonyls (PCO) (**B**), and malondialdehyde (MDA) (**C**) in the body wall of sea cucumber following exposure to elevated temperature (26 °C, light-blue filled), water-accommodated fractions (WAF) of Oman crude oil at an optimal temperature of 16 °C (WAF + 16 °C, light-red filled), and Oman crude oil WAF at an elevated temperature of 26 °C (WAF + 26 °C, light-yellow filled). The Control was the sea cucumber exposed to pre-filtered natural seawater only at an optimal temperature of 16 °C (light-green filled). Asterisks (*, **, or ***) denote the significant differences between the treatments and the Control (*p* < 0.05, 0.01, or 0.001, respectively). Dark traits denote the significant differences among the treatments (two-way analysis of variance (ANOVA)).

To assess the oxidative damage to macromolecules (DNA, proteins, and lipids) in body wall of sea cucumber following different treatments, the levels of 8-OHdG, PCO, and MDA were detected and are shown in Figure 5. For oxidative DNA damage (Figure 5A), results showed that the 8-OHdG levels were 2.29 ± 0.10 ng·mgprot^−1^, 2.08 ± 0.07 ng·mgprot^−1^, 2.50 ± 0.12 ng·mgprot^−1^, and 1.67 ± 0.07 ng·mgprot^−1^ for the 26 °C, WAF + 16 °C, WAF + 26 °C, and the Control groups, respectively, indicating that all the treatments caused a significant increase in 8-OHdG level compared to the Control (two-way ANOVA, *p* < 0.001, *p* < 0.01, and *p* < 0.001, respectively). The 8-OHdG level in the WAF + 26 °C group was 1.20-fold higher than that in the WAF + 16 °C group (two-way ANOVA, *p* = 0.008 < 0.01), while no significant differences were observed between the 26 °C group and the WAF + 26 °C group (two-way ANOVA, *p* > 0.05). As for the protein oxidation, PCO contents were detected in the present study (Figure 5B). Data showed that only WAF + 26 °C treatment caused a significant increase in the PCO content (6.23 ± 0.84 nmol·mgprot^−1^) compared to the Control (1.55 ± 0.31 nmol·mgprot^−1^, two-way ANOVA, *p* < 0.001), while the 26 °C and WAF + 16 °C treatments induced a slight rising trend but without significant differences (2.16 ± 0.75 nmol·mgprot^−1^ and 3.01 ± 1.32 nmol·mgprot^−1^, respectively; two-way ANOVA, *p* > 0.05). Furthermore, the MDA contents were also detected as a bioindicator of lipid peroxidation in the present study (Figure 5C). Differently, compared to the Control (0.67 ± 0.27 nmol·mgprot^−1^), all the treatments caused a significant elevation of MDA content, as evidenced by 1.82-, 2.07-, and 5.30-fold-higher values in the 26 °C, WAF + 16 °C, and WAF + 26 °C groups, respectively (two-way ANOVA, *p* < 0,05, *p* < 0.05, and *p* < 0.001, respectively).

### 3.5. Apoptosis Assessment

**Figure 6:**
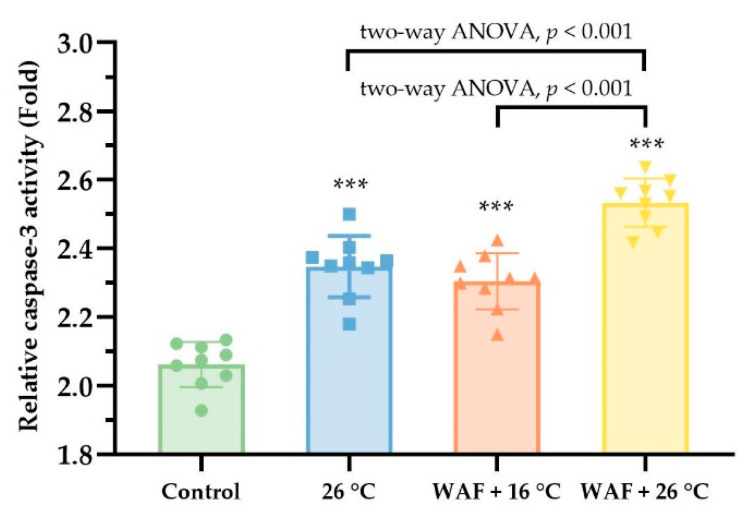
Relative caspase-3 activity (fold) in the body wall of sea cucumber following exposure to elevated temperature (26 °C, light-blue filled square), water-accommodated fractions (WAF) of Oman crude oil at an optimal temperature of 16 °C (WAF + 16 °C, light-red filled up-triangle), and Oman crude oil WAF at an elevated temperature of 26 °C (WAF + 26 °C, light-yellow filled down-triangle). The Control was the sea cucumber exposed to pre-filtered natural seawater only at an optimal temperature of 16 °C (light-green filled circle). Error bars represent 95% confidence intervals. Asterisks ***) denote the significant differences between the treatments and the Control (*p* < 0.001, respectively). Dark traits denote the significant differences among the treatments (two-way analysis of variance (ANOVA)).

To further evaluate the induction of cell apoptosis in the body wall of sea cucumber following different treatments, the relative changes of caspase-3 activity were measured as shown in Figure 6. The results showed that the relative caspase-3 activities in sea cucumber exposed to 26 °C, WAF + 16 °C, and WAF + 26 °C were 2.35 ± 0.03, 2.30 ± 0.05, and 2.53 ± 0.06, which were significantly higher than that in the Control (two-way ANOVA, *p* < 0.001). Moreover, the relative caspase-3 activities in the WAF + 26 °C treatment was much higher than that in the 26 °C and WAF + 16 °C treatments (two-way ANOVA, *p* < 0.001).

### 3.6. IBR Index

**Figure 7:**
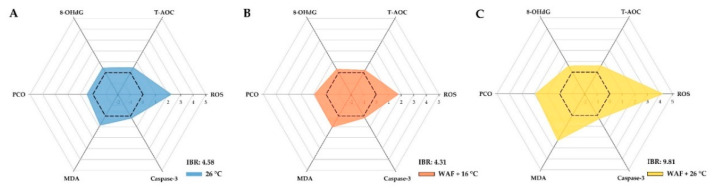
Integrated biomarker response (IBR) indexes for sea cucumber following exposure to elevated temperature (26 °C, light-blue filled, (**A**), water-accommodated fractions (WAF) of Oman crude oil at an optimal temperature of 16 °C (WAF + 16 °C, light-red filled, (**B**), and Oman crude oil WAF at an elevated temperature of 26 °C (WAF + 26 °C, light-yellow filled, (**C**). Biomarker results are denoted relative to the Control (sea cucumber exposed to pre-filtered natural seawater only at an optimal temperature of 16 °C) (black dash lines). The area above 0 denotes the induction of the biomarker, and the area below 0 denotes the reduction of the biomarkers. ROS: Reactive oxygen species; T-AOC: Total antioxidant capacity; 8-OHdG: 8-hydroxy-2′-deoxyguanosine; PCO: Protein carbonyls; MDA: Malondialdehyde.

The results of ROS, T-AOC, 8-OHdG, PCO, MDA, and caspase-3 were further analyzed through the IBR index to assess the global responses in the body wall of sea cucumber following the 26 °C, WAF + 16 °C, and WAF + 26 °C treatments, as shown in the star plots (Figure 7). The IBR indexes were 4.58, 4.31, and 9.81 for the 26 °C, WAF + 16 °C, and WAF + 26 °C treatments, respectively, indicating that the rank of the most affected groups could be ordered as WAF + 26 °C treatment > 26 °C treatment > WAF + 16 °C treatment.

## 4. Discussion

In the present study, we investigated the oxidative-related changes of sea cucumber to cope with acute exposure to crude oil at an elevated temperature of 26 °C, which exceeds the optimal temperature (16 °C) for sea cucumber inhabiting. Our results showed that the cellular ROS level, as a net content of ROS in vivo regulated by the equilibrium between the ROS production and scavenging, was much higher in sea cucumber exposed to the combination of elevated temperature and crude oil (WAF at 26 °C) than that exposed to elevated temperature alone (26 °C) or crude oil alone (WAF at 16 °C), as evidenced by 4.21- and 6.03-fold higher, respectively. It indirectly suggested that elevated temperature could markedly enhance the stimulation of ROS production in sea cucumber caused by crude oil exposure, resulting in a significant induced increase in the net ROS level. Consistent with our results, previous studies have also reported that significant increases in the ROS levels of marine organisms were recorded after exposure to elevated temperature [37,38,39] or crude oil [2,66,67]. As a natural byproduct of oxygen metabolism, ROS, such as hydrogen peroxide (H_2_O_2_), hydroxyl radical (OH), and superoxide anion (O^2−^), plays an integral role in normal cell signaling and cellular function [68,69]. However, environmental stresses could increase ROS production via affecting or interrupting respiratory electron transport chains, enzymatic reaction, and other normal metabolisms [70], which could subsequently alter intracellular redox homeostasis and cause cellular oxidative stress [31,39,69,71]. To maintain the redox homeostasis within certain limits, the antioxidant enzymatic system is developed by aerobic organisms as the primary defense line for ROS scavenging, such as superoxide dismutase (SOD) and catalase (CAT) [2,69,72]. In the present study, we evaluated the antioxidant defense capacity of sea cucumber following different treatments via the T-AOC. A significant increase in the T-AOC was observed in sea cucumber following exposure to elevated temperature alone or the combination of elevated temperature and crude oil relative to the Control, as evidenced by 1.40- and 1.53-fold-higher values, respectively. In contrast, crude oil alone exposure caused a subtle increase in the T-AOC (1.12-fold higher relative to the Control) but without a significant statistical difference. Recent studies have revealed that environmental stresses could stimulate ROS production in other marine benthos with a concomitant induced-increase in the capacity of antioxidant defense system to keep a degree of equilibrium between ROS production and scavenging [39,73,74]. Higher T-AOC observed in sea cucumber exposed to the combination of elevated temperature and crude oil than that in crude oil alone exposure might be related to the induced increase of ROS level caused by the co-exposure of the elevated temperature and crude oil. Moreover, a significant increase in the net ROS level observed in different treatments suggests that ROS production had exceeded the scavenging capacity of the antioxidant defense system in sea cucumber following different treatments, subsequently leading to the high abundance of ROS.

Excessive ROS could induce extensive oxidative damage to the biomacromolecules (e.g., DNA, proteins, and lipids), which is known as the primary mechanisms of toxic effects on marine benthos exposed to oil-derived PAHs [2,29,33]. In the present study, we detected 8-OHdG, PCO, and MDA levels in sea cucumber following different treatments to assess oxidative DNA damage, protein oxidation, and lipid peroxidation, respectively. The results showed that all the treatments resulted in various extents of increase in 8-OHdG, PCO, and MDA levels, indicating that exposure to elevated temperature, crude oil, or the combination of both could cause oxidative damage to the biomacromolecules of sea cucumber. Consistent with our study, other studies have reported that elevated temperature or crude oil exposure could induce severe oxidative damage of biomacromolecules in various marine organisms, e.g., ascidian (*Styela plicata*), polychaete (*Laeonereis culveri*), bivalve (*Anomalocardia flexuosa*), or sea cucumber (*Parastichopus regalis*) [29,75,76]. Additionally, we found that 8-OHdG, PCO, and MDA levels in sea cucumber following exposure to WAF at 26 °C were much higher than those following exposure to elevated temperature (26 °C) or WAF at 16 °C treatments, indicating that the co-exposure to elevated temperature and crude oil could cause much more severe oxidative damage to the macromolecules of sea cucumber than elevated temperature alone or crude oil alone did. Among the three biomarkers of oxidative damage, the change of MDA level in sea cucumber following different treatments was the most obvious, reinforcing the previous findings that lipid peroxidation is the relatively more sensitive biomarkers for the oxidative damage of the three main macromolecules in marine benthos under environmental stresses, especially oil-derived hydrocarbons exposure [29,33,77]. Furthermore, in the present study, we also found that relative to the Control, exposure to elevated temperature, crude oil, or the combination of both caused a significant increase in cellular apoptosis via caspase-3 activity, which is well established as one of the key executioners or final effectors for apoptosis to occur [78,79]. Cellular apoptosis is a physiological process by which the cell undergoes programmed death to eliminate redundant, unwanted, or damaged cells [74,79,80]. It is generally known that cellular apoptosis naturally occurs during cell development and aging and is a crucial survival mechanism for marine organisms under environmental stresses [29,73]. Our results supported previous findings that severe cellular apoptosis is another obvious secondary response to the overproduction of ROS in marine benthos induced by environmental stresses, which has been suggested to involve the oxidative damage of biomacromolecules, especially oxidative DNA damage [81,82]. Severe cellular apoptosis might consequently result in reducing the survival of marine benthos or even threatening their population [73].

As different biochemical markers showed different sensitivity to environmental stresses, we employed the IBR index that integrated all the biochemical responses into one index to provide a quantitative expression of the combined biochemical effects of the six biomarkers in sea cucumber following different treatments. The results showed that the IBR index of sea cucumber exposed to the combination of elevated temperature and crude oil was much higher than that exposed to elevated temperature or crude oil, which was mainly attributed to the higher induction of ROS level and MDA content. Previous studies have reported that elevated temperature could increase reaction rates as well as metabolism, which may in turn increase the sensitivity of marine organisms to oil pollution [25,26]. This increased sensitivity could be one of the main reasons for interpreting that the co-exposure to elevated temperature and crude oil caused much severer toxic effects on sea cucumber than elevated temperature or crude oil did. Moreover, our chemical analysis revealed that the levels of TPH and ΣPAHs in WAF solution at 26 °C were 1.10- and 1.26-fold higher than those in WAF solution at 16 °C, especially for HMW PAHs (≥3-ringed) that showed a much higher proportion in WAF solution at 26 °C than that in WAF solution at 16 °C. It has been accepted that HMW PAHs are relatively strong agonists of the aryl hydrocarbon receptor (AhR) [83]. Recently, novel studies have documented that PAHs metabolism and detoxification in marine benthic invertebrates similar to the mammalian or fishes are also complex processes involved with the AhR signaling pathway, which could generate a large number of active PAHs metabolic intermediates and ROS substances [84,85]. Consistent with our study, other studies on fish species have also found that elevated temperature could acutely increase the contents of dissolved hydrocarbons from crude oil into the water column to a certain extent [44,45], which subsequently could increase the bioavailability of oil-derived hydrocarbons and lead to much more toxic effects on marine organisms [45]. We inferred that the increase of dissolved hydrocarbons caused by elevated temperature could be another main reason for the much severer toxic effects observed in sea cucumber following co-exposure to elevated temperature and crude oil.

## 5. Conclusions

Overall, in the present study, we explored the combined effects of elevated temperature and crude oil exposure on oxidative stress and apoptosis in the body wall of sea cucumber. Our results revealed that elevated temperature enhanced the stimulation of ROS production and its secondary responses related to oxidative stress caused by crude oil to sea cucumber. We speculated that elevated temperature could increase PAHs metabolism in sea cucumber and the sensitivity of sea cucumber to crude oil. Besides, elevated temperature also should attribute to the higher levels of dissolved hydrocarbons from crude oil into water column, subsequently increasing the bioavailability of sea cucumber to crude oil. Therefore, our results suggested that comprehensive consideration of the environmental conditions is necessary for oil toxicity testing and its risk assessments. Future research should further clarify the underlying molecular mechanisms involved in oxidative damage and apoptosis in sea cucumber following exposure to oil-derived hydrocarbons at elevated temperatures.

## Data Availability

Data available on request due to restrictions eg privacy or ethical.
